# GazePlotter: An open-source solution for the automatic generation of scarf plots from eye-tracking data

**DOI:** 10.3758/s13428-026-02959-5

**Published:** 2026-03-12

**Authors:** Michaela Vojtechovska, Stanislav Popelka

**Affiliations:** Department of Geoinformatics, Palacký University Olomouc, 17. listopadu 50, 779 00 Olomouc, Czechia

**Keywords:** Eye-tracking, Scarf plot, Visualisation tool, Open source

## Abstract

Eye-tracking is widely used to study perception, learning, and decision-making, yet visualising temporally structured gaze behaviour remains challenging. Scarf plots (also known as sequence charts) help illustrate when and where participants focus attention, but existing tools are often proprietary, static, or require programming expertise. GazePlotter is an open-source, browser-based application designed to lower these barriers. GazePlotter automatically processes raw exports from six eye-tracking software tools, including Tobii Pro Lab and GazePoint Analysis, and generates interactive, customisable scarf plots. It supports dynamic areas of interest (AOIs), multiple timeline modes (absolute, relative, ordinal), and enables side-by-side comparisons across participants, groups, or stimuli. Complementary visualisations—such as bar charts and transition matrices—can be combined within interactive dashboards. The application runs entirely in the browser (available at https://gazeplotter.com), preserving data privacy and requiring no installation or registration. Its data pipeline—from import through parsing, AOI aggregation, and export—was validated against proprietary software outputs, with high agreement across key metrics. Parsing is memory-efficient and tested on multi-gigabyte datasets, with consistent functionality across Chrome, Firefox, Safari, and Edge. A task-based usability evaluation demonstrated successful task completion and positive perceived pragmatic and hedonic quality among participants with prior experience in eye-tracking methodology. By enabling non-programmers to create exploratory visualisations directly from raw exports, GazePlotter makes temporally structured gaze data accessible for reproducible, shareable visual analysis. Beyond eye-tracking, the same workflow generalises to any temporally ordered categorical sequence from a compatible CSV input.

**Supplementary Information:**

The online version contains supplementary material available at 10.3758/s13428-026-02959-5.

## Introduction

Eye-tracking is a non-invasive measure of overt visual attention that provides high-resolution spatio-temporal data on how individuals engage with stimuli (Carter & Luke, 2020). It is widely used across fields concerned with perception, learning, and decision-making—including psychology, education, and user experience research (Goldberg & Kotval, 1999; Jarodzka, Holmqvist, & Gruber, 2017; Mele & Federici, 2012)—to study cognitive processes, attentional dynamics, and user interaction in both laboratory and field settings.

Interpreting attention from eye movement data often begins with heatmaps of gaze dwell distribution, favoured for their operational simplicity (Blascheck et al., 2017; Bojko, 2009; Hooge et al., 2025). However, Bojko (2009) warns that their intuitiveness is deceptive. By compressing gaze into static fields, typical heatmaps introduce temporal flattening, which erases the underlying cognitive strategy. Without a chronological dimension, researchers cannot distinguish early orienting from late-stage evaluation. Furthermore, spatial smoothing masks individual variability; a single outlier’s intense interest can propagate as a spurious group consensus, presenting a false representation of collective attention. While temporal encodings like scanpaths recover chronological order, they scale poorly for large cohorts (Blascheck et al., 2017). Separately, in time-varying stimuli (e.g., video), reference-frame instability often invalidates coordinate-based visualisations entirely, as raw *x*, *y* coordinates lose their semantic meaning when the underlying objects move (Kurzhals & Weiskopf, 2013).

*Areas of interest (AOIs)* discretise the visual field into functional units, mapping raw gaze coordinates onto semantic categories. Although this approach is sensitive to boundary definition (Hessels, Kemner, van den Boomen, & Hooge, 2016; Orquin, Ashby, & Clark, 2016), it enables the use of *dynamic AOIs*, which mitigate reference-frame instability by anchoring regions to time-varying stimulus elements (e.g., moving objects or interaction-dependent interface components; Friedrich, Rußwinkel, & Möhlenbrink, 2017; Mercier, Ertz, & Bocher, 2024; Röhrl, Hauser, Ezer, Grabinger, & Mottok, 2025). Most analyses collapse these data into aggregate summaries—such as total dwell time—which, while suitable for inferential statistics, effectively reintroduce an erasure of the spatio-temporal trajectory of attention. This loss of granularity is particularly problematic in qualitative or process-oriented studies, where the sequence and rhythm of information acquisition are more critical than cumulative exposure.

*Scarf plots* (also known as sequence charts), introduced by Richardson and Dale (2005), represent gaze as colour-coded AOI segments aligned on a timeline and stacked by participant (Blascheck et al., 2017). Scarf plots are particularly informative for answering questions about when participants attend to specific elements and who does so (Claus, Hermens, & Bromuri, 2025), although interpretability may decrease as the number of AOIs grows (Yang & Wacharamanotham, 2018). Alongside techniques like transition matrices or sequence graphs, scarf plots reveal shared strategies, anomalies, and group differences, supporting exploratory and comparative analysis.

In practice, however, producing sequence visualisations—such as scarf plots—especially when handling dynamic AOIs, remains nontrivial. As in many eye-tracking workflows, even when proprietary software is available, the work typically requires coding in R, MATLAB, or Python (Blascheck et al., 2017), yielding largely static figures. This lack of interactivity creates a significant barrier to rapid gaze inspection, cross-condition comparison, and the iterative refinement of qualitative interpretations. To bridge this gap for both experts and beginners, we developed GazePlotter: an open-source, browser-based tool that enables accessible, interactive exploration of AOI sequences without the need for scripting.

### Existing software for scarf plot generation

The lack of accessible tools for interactive exploration is especially evident in the case of scarf plots—a method well-suited for analysing temporally structured AOI sequences, yet rarely implemented in available platforms. Of proprietary software offered by eye-tracker manufacturers, only the discontinued SMI BeGaze allows for their generation. Software such as Tobii Pro Lab and GazePoint Analysis do not offer this option. In addition, we are only aware of SEQIT (Wu & Munzner, 2014), AlpScarf (Yang & Wacharamanotham, 2018), and GazeAlytics (Chen et al., 2023) as open-source solutions capable of generating scarf plots from eye-tracking data.

SEQIT is a JavaScript application built on top of the D3.js library, and it allows for the generation of simple scarf plots with an absolute and a relative timeline, accompanied by the visualisation of scan paths (Wu & Munzner, 2014). In comparison, AlpScarf is a Shiny application based on the R language, which permits the construction of typical scarf plots and their enriched versions, called alp scarfs. Alp scarfs encode information about the conformity of AOI visit order and revisits directly into the visualisation (Yang & Wacharamanotham, 2018). GazeAlytics differs from SEQIT and AlpScarf by focusing not only on scarf plot-like visualisation but also on other AOI analysis capabilities. Besides its relatively simple scarf plots, this web-based JavaScript and Python tool offers various visualisations of AOI transitions, scan paths, and others (Chen et al., 2023).

Despite their valuable contributions to the eye-tracking field, these open-source tools, as well as SMI BeGaze, exhibit significant limitations for comprehensive scarf plot analysis:

- *Data interoperability issues*: All reviewed tools require custom data formats or preprocessing pipelines. AlpScarf accepts only its own upload format (Yang & Wacharamanotham, 2018), GazeAlytics requires prior transformation into its structured layout (Chen et al., 2023), and SMI BeGaze—now deprecated—only supports its proprietary experiment files. SEQIT, a demo application, does not support file uploads at all (Wu & Munzner, 2014). These requirements impose additional work on users, often necessitating the creation of custom scripts. This introduces barriers for researchers without programming skills and increases the risk of errors or data loss during conversion.
- *Restricted support for dynamic AOIs*: None of the existing tools fully support AOIs that change position or visibility over time. SMI BeGaze partially visualises AOI visibility, but only for a single participant, limiting its utility for comparative analysis. GazeAlytics, AlpScarf, and SEQIT do not support dynamic AOIs at all. Without the ability to represent AOI dynamics, researchers cannot analyse how gaze interacts with time-varying stimuli, such as videos, animations, or interactive interfaces.
- *Limited visualisation customisation*: While GazeAlytics provides some flexibility for AOI analysis, its scarf plot visualisations are limited in scope. Eye movement types, such as saccades and fixations, cannot be visually distinguished. Similar constraints apply to SMI BeGaze, SEQIT, and AlpScarf. None of the tools support plotting multiple scarf charts in parallel—for instance, to compare participant groups, stimuli, or timeline types (absolute, relative, ordinal)—within a shared visual frame.
- *Privacy concerns*: AlpScarf and GazeAlytics require data to be processed on remote servers. This raises privacy and compliance concerns, particularly when handling sensitive eye-tracking datasets in clinical, educational, or institutional contexts. As eye-tracking data can reveal cognitive states or mental health indicators (Kröger, Lutz, & Müller, 2020), local processing is preferable for maintaining control and confidentiality, and may be required by institutional policies in some instances.

### Design requirements for an accessible scarf plot platform

GazePlotter was developed in close collaboration with future users, with many of its requirements emerging gradually in response to recurring issues in existing scarf plot tools. While not fixed from the outset, the design consistently aimed for accessibility, ease of use, and local control over data—aligning with user-centred design principles outlined in ISO 9241-210 (International Organization for Standardization, 2019). The list reflects the main design priorities:

- *Export compatibility*. Accept exports from major eye-tracking software tools (e.g., Tobii Pro Lab, BeGaze) without requiring format conversion or scripting.
- *Barrier-free access*: Operate fully in the browser, with no installation, registration, or payment required.
- *Dynamic AOI handling*. Represent AOIs that change in position or visibility over time, visualising when each AOI is visible for each participant.
- *Advanced visualisation control*. Enable users to customise visual encodings and interactively filter or sort data views.
- *Client-side data processing*: Perform all computations locally to preserve privacy and institutional compliance.
- *Open-source codebase*. Develop the tool under an open-source licence in a public code repository to facilitate transparency and community contributions.
- *Configurable comparison views*. Enable side-by-side alignment of scarf plots across participants, groups, stimuli, or time structures.
- *Complementary AOI visualisations*. Support the integration of additional visualisation types—such as transition matrices—into the main analysis view.

## Methods

To meet these goals, we developed GazePlotter as a client-side, open-source Progressive Web App for accessible, flexible scarf plot generation and other AOI-based gaze visualisations. This paper focuses on 1.7.x release cycle: technical validation was established on version 1.7.1, while the user study and feature description use version 1.7.6. This transition introduced user interface refinements, stability fixes, and expanded data format support, while the validated core parsing logic remained identical across both versions. Here, we outline (1) the system’s architectural evolution, (2) the testing strategy, and (3) the role of user feedback in shaping interface design and usability, along with the current state of documentation.

### Development process

The initial prototype (Vojtechovska & Popelka, 2023)—built entirely in modular TypeScript without a frontend framework—proved difficult to extend as interface complexity increased. To address this, we restructured the application with SvelteKit and configured it for static site generation, enabling cleaner separation between interface logic, state management, and core data operations. Svelte stores manage dashboard-level state, including individual gaze visualisations’ position, dimensions, and filtering parameters. Svelte components consume this state to render interactive visual elements—such as scarf plots or transition matrices—and coordinate user actions like dragging panels or selecting data subsets. Core data operations, including querying from a lightweight in-memory gaze data structure and applying transformations, are delegated to client-side TypeScript modules.

GazePlotter is currently built with SvelteKit 2.26.1 (Svelte Contributors, 2018), using Svelte 5.36.17 (Svelte Contributors, 2016) and TypeScript 5.8.3 (Microsoft, 2012). Parsing and transformation routines are encapsulated in TypeScript classes, decoupled from the interface, and executed in parallel via Web Workers to prevent the main thread from blocking. Rather than loading entire files into memory, the system processes data incrementally by reading one chunk at a time using the JavaScript ReadableStream API. The streaming mechanism ensures stability even on low-performance devices or with multi-gigabyte files—processing time increases with file size, but memory usage remains bounded.

The final build is a Progressive Web App (PWA) compiled to static assets, compatible with all major browsers on desktop and mobile platforms. Deployment and testing are automated via GitHub Actions. Source code, issue tracking, and contribution guidelines are openly maintained at: https://github.com/misavojte/GazePlotter. During later phases, GitHub Copilot, an AI-based assistant powered by a generative language model, was used for low-level code completion.

### Testing and validation strategy

To ensure correctness, robustness, and maintainability, *GazePlotter* was evaluated through multiple strategies: (1) exploratory inspection during parser development, (2) systematic unit testing, (3) cross-browser end-to-end testing, and (4) metric-level validation against proprietary software. Each method addressed a different layer of potential error, from low-level parsing quirks to user-facing interface inconsistencies and high-level metric discrepancies, forming a layered validation pipeline. Sample eye-tracker exports are available in an open repository (Vojtechovska & Popelka, 2025).

1. *Exploratory inspection during parser development.* Support for each new data format began with hands-on inspection of raw parsed outputs. These reviews—conducted across all supported formats—involved checking the first, last, and several randomly sampled gaze segments per participant to identify format-specific irregularities. This approach revealed subtle but critical edge cases, including off-by-one segment boundaries, ambiguous timestamp alignment, and overlapping AOI assignments. While some of these issues could surface in aggregate metrics, direct contact with event-level data enabled faster identification and clearer resolution. Many of these edge cases were later formalised into automated tests to ensure continued reliability.
2. *Unit testing.* Insights from exploratory inspection were translated into structured unit tests targeting the most critical components of the transformation pipeline. Unit testing refers to the process of verifying individual software modules in isolation to ensure consistent behaviour across a range of inputs, enabling regression detection and preserving correctness as the codebase evolves (Zhu, Hall, & May, 1997). We implemented the suite using the Vitest 1.1.3 framework (Fu & Capeletto, 2021), selected for its compatibility with the Vite and SvelteKit stack. Given the limited size of the core development team, test coverage is necessarily selective and focused on high-impact functionality rather than comprehensive implementation, leaving some lower-risk paths untested.
3. *Cross-browser end-to-end testing.* To ensure consistent functionality across environments, we evaluated *GazePlotter*’s interface and interactive components across major browsers—including Chrome, Firefox, Safari, and Edge (on Windows, macOS, iOS, and Android)—to verify interaction fidelity and visual consistency. Testing focused on core user operations such as data import, scarf plot rendering, navigating and repositioning visualisations within the dashboard, modifying AOIs, and exporting results. Basic performance metrics, including memory usage and parsing time, were monitored using the GazePlotter’s metadata panel to assess behaviour across browsers and datasets. All tests were conducted using real data from various eye-tracking systems and software exports (see Table 1), reflecting the range of formats supported by the platform.
4. *Validation against proprietary software.* To confirm that *GazePlotter* produces outputs consistent with established standards, we conducted formal validation of aggregated AOI metrics—time to first fixation, average fixation duration, and fixation count—against exports from Tobii Pro Lab and SMI BeGaze. We chose these metrics for their ubiquity in AOI-based workflows and their sensitivity to parsing and alignment errors. For each AOI-participant combination in a real-world dataset, we compared GazePlotter’s computed values to those of the proprietary software using strict tolerances: ±1 ms for temporal metrics and exact matches for counts. This benchmarking served as an external check on system-level correctness.

**Table 1:** Used data formats of various software and eye-trackers for GazePlotter end-to-end testing

Software	Tested eye-trackers
SMI BeGaze	SMI RED 250 eye-tracker
Tobii Pro Lab	Tobii Pro Spectrum 300
	Tobii Pro Glasses 3
GazePoint Analysis	GP3 HD Eyetracker
	GP3 Eyetracker
OGAMA	GP3 HD Eyetracker (GazePoint Analysis conversion)
	Tobii Pro Spectrum 300 (Tobii Pro Lab conversion)
Varjo XR	Varjo XR-4 (virtual reality headset)
Pupil Cloud	Pupil Labs Neon
MS Excel	Custom sequential non-gaze data

**Fig. 1:**
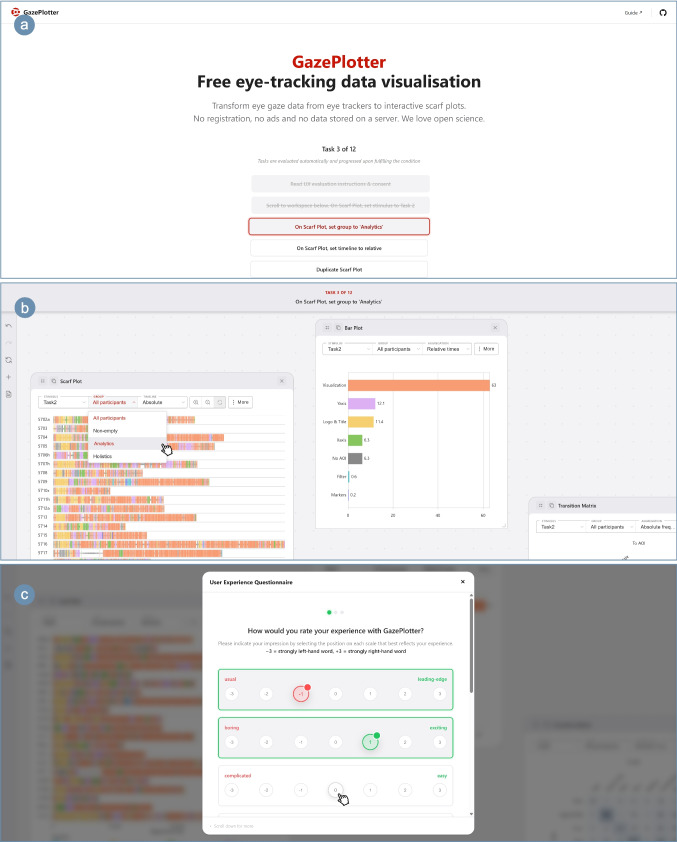
Screenshots of built-in UX study on 1920x800px viewport: **a**) task list on top of the page, **b**) active workspace with task banner after scrolling down, and **c**) questionnaire pop-up

**Fig. 2:**
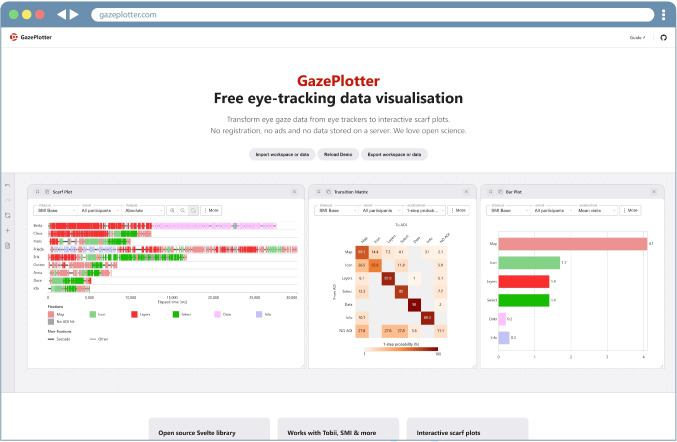
View of the GazePlotter interface with pre-loaded demo data in a web browser. The workspace holds one scarf plot, transition matrix, and bar plot

### User experience

The development of GazePlotter followed an iterative, user-centred design process. Initial interface design was guided by informal academic feedback gathered during presentations at major eye-tracking conferences, including the Eye Tracking Research and Applications Symposium (ETRA 2024, ETRA 2025) and the European Conference on Eye Movements (ECEM 2024). This feedback from researchers—particularly those with limited programming experience—informed the tool’s progression from a single scarf plot viewer to a multi-view dashboard supporting transition matrices and aggregation charts.

Documentation was developed alongside the system to support both contributors and end users. Developer-facing comments follow the JSDoc standard, while user guidance is provided via an interactive manual available at docs.gazeplotter.com. The guide is authored in Markdown using VitePress 1.0.0-rc.44 (You, 2019) for fast contributions.

To evaluate user experience among participants not involved in earlier feedback cycles, we conducted a remote, instrumented usability evaluation integrated into GazePlotter’s interface (see Fig. 1 for the study environment). Forty-one usability sessions were collected; six were excluded due to early termination, leaving 35 participants for analysis. The sample comprised European eye-tracking researchers recruited through targeted outreach ($n=18$) and students already familiar with eye-tracking at Palacký University Olomouc, Czech Republic ($n=17$; six international); participation was voluntary and uncompensated.

Following GDPR requirements and institutional ethical approval, the study employed a 12-step protocol comprising (a) informed consent, (b) ten instrumented functional tasks performed on a pre-loaded dataset, and (c) a survey including an expertise question, the Short User Experience Questionnaire (UEQ-S; Schrepp, Hinderks, & Thomaschewski, 2017), and open-ended textual feedback. Task success and completion times were logged in real time via application-level events in the system’s undo/redo pipeline. All evaluation components are provided as Supplementary Materials: instrumentation logic is included in the source code (under src/survey), while the Python anonymisation and aggregation pipeline, the anonymised dataset, and the populated *Short UEQ Data Analysis Tool v13* (Schrepp, 2025)—from which UEQ-S metrics and benchmark comparisons were derived—are provided separately.

## Results: GazePlotter app

The GazePlotter web application (Fig. 2) addresses long-standing limitations in scarf plot generation by providing an accessible, browser-based platform for AOI-based sequence visualisation. Available at gazeplotter.com, it operates without installation, login, or external data processing, and supports direct import of raw exports from multiple eye-tracking software tools. This section outlines five core outcomes of the development effort: (a) the tool’s key features, (b) an illustrative walkthrough demonstrating a typical analysis session, (c) a comparison with existing scarf plot solutions, (d) results from internal testing and validation, and (e) real-world use cases of the application in both laboratory and applied research contexts.

### Features

GazePlotter provides a browser-based environment for parsing, visualising, and exporting AOI-based gaze data. The features below reflect version 1.7.6, with up-to-date workflows and technical details available in the documentation (docs.gazeplotter.com).

1. *Data handling and format support:* GazePlotter accepts exports from six eye-tracking software tools (SMI BeGaze, Tobii Pro Lab, OGAMA, GazePoint Analysis, Varjo XR, and Pupil Cloud), automatically recognising formats by internal structure. Where the stimulus assignment is ambiguous, users are prompted to resolve it.
  In addition to native formats, GazePlotter supports plain CSV input in two layouts, enabling use with third-party trackers, custom pipelines, or any sequential data—eye-tracking or not—containing temporally ordered events.
  Once parsed, users can edit data directly within the interface. Users may merge or recolour AOIs, rename stimuli, and group participants non-destructively. Parsing metadata—including filename, detected format, file size, and duration—is saved with the session alongside the GazePlotter version, supporting auditability.
2. *Advanced scarf plots:* Scarf plots serve as the primary visualisation, depicting AOI-labelled sequences for each participant. Rows represent individual participants; segments are coloured by AOI, with optional split rendering for overlaps. Hover interactions reveal segment-level metadata, including AOI name, time range, and duration.
  To support different interpretive goals, users can switch between three timeline modes: absolute (milliseconds), relative (normalised duration), and ordinal (segment order). To support the analysis of dynamic AOIs, an optional visibility layer plots when AOIs are visible to the participant over time using colour-coded bars, configurable globally or per participant.
  All scarf plots are filterable by stimulus, participant group, AOI, or time range, with filters applied non-destructively and reversible at any time.
3. *Supplementary visualisations:* GazePlotter supports programmatic extension with custom AOI-based visualisations. Two types are included by default:
  *Bar plots*, which summarise dwell time, fixation count, and time to first fixation.
  *Transition matrices*, which visualise AOI-to-AOI transitions as heat-mapped adjacency matrices.
  Both plot types support interactive tooltips, participant or stimulus-level filtering, and customisable settings for exploratory analysis or presentation.
4. *Shareable interactive dashboard:* All visualisations are embedded in a dashboard that supports drag-and-drop layout control. Users may duplicate, resize, remove, or arrange views freely, creating multi-panel setups with different filters and configurations.
  Dashboards can be saved as lightweight JSON files containing all loaded data, layout states, and settings. These exports are cross-device compatible, version-tracked, and reproducible—enabling collaboration or continued analysis without reprocessing the original data.
5. *Data Export:* Each visualisation can be exported as a raster image in PNG, JPG, or WebP format, with configurable DPI and margins. All exports preserve the current view state to ensure visual consistency for publication.
  Structured CSV exports include both raw event data (e.g., fixations, saccades) and derived AOI-level metrics. A dedicated export function also generates ScanGraph-compatible (Dolezalova & Popelka, 2016) files for graph-based scanpath comparison without additional formatting.
6. *Privacy, offline use, and distribution:* GazePlotter runs fully client-side. All data are processed locally in the browser’s memory, never transmitted externally, and not retained beyond the session, supporting use in privacy-sensitive settings such as clinical or educational research. The only exception was the instrumented usability evaluation, for which we collected pseudonymised interaction logs and questionnaire responses only after obtaining full informed consent.
  As a Progressive Web App (PWA), it supports full offline functionality across devices. The tool is open-source (GPLv3), with modular components available via GitHub^1^. In addition, GazePlotter’s core components are published as modular npm packages, allowing developers to reuse its visualisation modules in custom Svelte-based environments.

### Illustrative walkthrough

To illustrate the practical use of GazePlotter, we revisit a study by Popelka, Burian, & Beitlova (2022), which involved analysing gaze behaviour on dynamic web map applications. In the original workflow, the researchers used SMI BeGaze to export participant-level AOI data. However, due to the software’s limitation of only displaying one participant at a time and lacking support for dynamic AOI visibility, the team was forced to manually assemble sequence graphs in Adobe Photoshop—an effort that required several hours of repetitive editing per dataset. We replicated the desired scarf plots in GazePlotter in several minutes using the following workflow:

1. *Export data from SMI BeGaze.* AOI sequence data and AOI visibility information are exported using the standard BeGaze export functions. The AOI visibility files are exported separately.
2. *Upload eye-tracking data to GazePlotter.* The exported participant data file is selected via the upload button in the browser interface at https://gazeplotter.com. The application automatically detects the format and parses stimulus, participant, and AOI information.
3. *Assign AOI visibility file.* The user selects the “AOI visibility” option from the scarf plot menu. In this case, each participant interacted with a dynamic web application, where AOIs were activated and deactivated based on individual interface use. As such, a separate AOI visibility file was assigned to each participant individually. In contrast, for video-based stimuli where visibility is consistent, the same visibility file could be applied globally to all participants.
4. *Customise scarf plot appearance.* AOIs are renamed and recoloured within the interface to improve legibility. AOIs can be merged and unmerged. The timeline axis is switched from absolute to relative mode to normalise differences in viewing duration across participants.
5. *Group participants for comparison.* Participants are assigned to groups (e.g., “novice” vs. “expert”) via the group editor. Separate scarf plots are created for each group using filters and positioned side by side in the dashboard.
6. *Adjust layout and inspect patterns.* The scarf plots are rearranged and resized via drag-and-drop. Users inspect segment-level detail using tooltips and assess dynamic AOI presence using the visibility bars below each row.
7. *Add bar plot for aggregated AOI metrics.* To supplement the sequence-level view, the user adds a bar plot visualisation showing aggregated metrics (e.g., total dwell time and fixation count) for each AOI across participant groups. This view provides a quick comparison of overall attention allocation and complements the temporal structure shown in the scarf plots.
8. *Export visualisations and metrics.* Scarf plots and bar plots were exported as PNGs at print-quality resolution (600 DPI), and AOI-level metrics were saved as CSV for statistical analysis. GazePlotter also provided direct export to the ScanGraph tool (Dolezalova & Popelka, 2016), enabling direct scanpath comparison in ScanGraph without the need for manual reformatting.
9. *Share dashboard with collaborators.* The session JSON file containing all loaded data, layout settings, and visualisation configurations was sent to collaborators, who were able to open it directly in GazePlotter on their own devices. This enabled distributed review, ensured reproducibility, and avoided the need to reprocess the original dataset.

### Comparison with other scarf plot solutions

Table 2 compares GazePlotter with selected existing tools that support scarf plot generation. While many of these platforms offer broader analytical functionality, the comparison is limited to scarf plot-related features to highlight differences in timeline control, input compatibility, and support for dynamic AOIs. GazePlotter addresses several limitations observed in earlier tools, including limited input format compatibility, lack of support for dynamic AOIs across participants, and constrained visual customisation. It integrates automated data recognition, support for multi-format and multi-participant input, and timeline flexibility (supporting absolute time, relative time, and ordinal sequence views), while maintaining accessibility through a browser-based interface with no installation and programming required.

**Table 2:** Feature comparison of scarf plot tools

Criteria	GazePlotter	BeGaze	GazeAlytics	AlpScarf	SEQIT
Data Upload					
Multiple sources	Yes	No	No	No	No
Local processing	Yes	Yes	No	No	No
Dynamic AOIs					
Single participant	Yes	Yes	No	No	No
Multiple participants	Yes	No	No	No	No
Scarf plot capabilities					
Show >1 eye movement types	Yes	No	No	No	No
Multiple scarf views	Yes	No	No	No	No
Segment metadata on hover	Yes	No	No	No	Yes^a^
Absolute, relative, and ordinal	Yes	No	No^b^	No^b^	No^b^
timeline					
Dynamic Analysis Control					
Filter by participant	Yes	Yes	Yes	Yes	No
Filter by stimulus	Yes	Yes	Yes	No	No
Participants group/rename	Yes	Yes	Yes	No	No
AOIs group/rename/recolor	Yes	Yes	Yes	No	No
Drag-and-drop layout control	Yes	No	No	No	No
Other visualisation types	Yes	Yes	Yes	Yes	Yes

^a^ Displays AOI label and visit order; does not expose temporal extent or duration of segments.

^b^ Supports absolute and relative time, but lacks an ordinal sequence mode

### Testing and validation results

To confirm that GazePlotter performs consistently across environments and dataset sizes, we conducted end-to-end interface tests and parsing trials using real-world data. Interface components were verified across Chrome, Firefox, Safari, and Edge for visual and interaction fidelity. Core operations—including data import, scarf plot rendering, AOI assignment, dashboard navigation, and export—functioned reliably in all cases. Performance metrics, including memory usage and parsing duration, were recorded using GazePlotter’s internal diagnostics.

To illustrate practical scalability, we parsed two datasets of increasing complexity. A medium-sized export (132 MB, 26 participants, six AOIs) was processed using the Tobii parser—the most complex in GazePlotter due to extensive branching logic required for mobile eye-tracking exports and nonstandard timestamp structures. On a mid-range laptop (HP ProBook 455 G6, AMD Ryzen 5 2500U @ 2.00 GHz, 8 GB RAM, Windows 10 Enterprise 22H2), this completed in 1.45 s with heap memory remaining under 17 MB. A substantially larger file (12.76 GB, 38 participants, 232 stimuli, 1320 AOIs) was parsed in 3 min and 43 s on the same device, with heap usage staying below 30 MB throughout. These sessions can be exported as shareable dashboards with three active visualisations, reducing storage requirements to 340 KB and 5.6 MB respectively.

As shown in Table 3, GazePlotter’s AOI-level metrics closely matched those exported from *SMI BeGaze 3.6.57* and *Tobii Pro Lab 1.241*. For BeGaze, fixation counts and time-to-first-fixation values exhibited exact matches across all AOI–participant combinations. Deviations in average fixation duration were minimal (mean difference –0.001 ms), attributable to rounding artifacts in BeGaze’s exports.

**Table 3:** Validation of GazePlotter outputs against proprietary software. Values represent differences between GazePlotter and reference outputs in fixation metrics (GazePlotter minus original), aggregated across AOI-participant combinations

Statistic	Time to First Fix. (ms)	Avg. Fix. Duration (ms)	Fix. Count
*SMI BeGaze 3.6.57*			
Min	0	-0.100	0
Max	0	0.054	0
Mean	0.000	-0.001	0.000
SD	0.000	0.023	0.000
*Tobii Pro Lab 1.241*			
Min	-0.735	-0.489	0
Max	0.685	0.643	0
Mean	0.006	-0.062	0.000
SD	0.287	0.276	0.000

In Tobii Pro Lab, fixation counts again matched exactly, while small differences were observed in temporal metrics (mean difference 0.006 ms for time to first fixation; –0.062 ms for average fixation duration). These stem from Tobii’s export structure, which rounds timestamps to full milliseconds and divides data into multiple AOI—participant intervals—a structure common in mobile eye-tracking, where stimuli can reappear. For comparison, we reconstructed GazePlotter metrics across these intervals to produce unified AOI–participant values. This reconstruction introduced minor compounded offsets due to rounding mismatches. However, even the largest recorded deviations remained within the pre-defined ±1 ms tolerance, demonstrating that both the parsing logic and AOI-level aggregation operate as intended when compared to proprietary software.

**Fig. 3:**
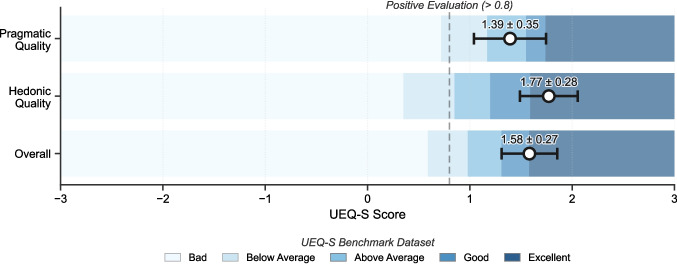
UEQ-S scale scores for GazePlotter relative to the global benchmark dataset. Error bars represent the 95% confidence interval

### User evaluation

Following the iterative development phase informed by practitioner feedback, testing, and technical validation, we assessed GazePlotter’s practical utility through a usability evaluation. Participants ($N = 35$) attempted all ten in-app tasks in a mean duration of 501.53 s ($SD = 166.10$ s), excluding the time allocated for instructions, informed consent, and questionnaires. 33 participants completed all tasks, while two participants skipped a single operation—specifically the AOI grouping operation nested within the advanced AOI settings—but successfully finished the remaining nine.

The UEQ-S scale ranges from -3.0 (extremely negative) to +3.0 (extremely positive), with values above 0.8 indicating a positive evaluation (Schrepp et al., 2017). Item-level analysis revealed the lowest means and greater variability for item 4 (confusing vs. clear; $M = 1.0, SD = 1.7$) and item 2 (complicated vs. easy; $M = 1.2, SD = 1.5$).

Open-ended feedback further contextualised these findings. Negative comments focused primarily on the pannable workspace ($n=7$), where duplicated plots rendered outside the active viewport on smaller displays were occasionally overlooked. Some users reported difficulties with AOI grouping ($n=4$), and expert participants noted that the lack of contextual information about the pre-loaded eye-tracking dataset limited their ability to immediately interpret the visualisations ($n=3$).

Despite these initial challenges, the overall UEQ-S results indicate a high level of user satisfaction across both dimensions (Fig. 3). The Pragmatic Quality scale achieved an “Above average” rating ($M = 1.39, SD = 1.07, \alpha = 0.78$), indicating that GazePlotter outperformed more than 50% of products in the benchmark dataset. The Hedonic Quality scale reached the “Excellent” category ($M = 1.77, SD = 0.85, \alpha = 0.77$), placing the tool among the top 10% of evaluated products globally.

The strongest item-level ratings were observed for item 6 (not interesting vs. interesting; $M = 2.0, SD = 0.9$) and item 3 (inefficient vs. efficient; $M = 1.9, SD = 1.0$), highlighting both engagement and perceived efficiency. The Overall evaluation yielded a mean score of 1.58 ($SD = 0.82$) with a 95% confidence interval of [1.31, 1.85], which is categorized as “Excellent” relative to the global benchmark. Internal consistency exceeded the 0.7 threshold for both scales, confirming reliable interpretation of questionnaire items.

Positive open-ended responses ($n=28$) aligned with the quantitative findings, most frequently praising the visual design ($n=9$) and the quick responsiveness of the interface ($n=5$), reinforcing the strong hedonic and pragmatic evaluation of GazePlotter.

### Case studies

GazePlotter has already been applied in a variety of published and ongoing research projects. The following case studies, grouped by research objective, reflect a subset where we have direct access to original data. Additional uses exist; for the examples below, data can be requested from the corresponding author.

#### Evaluation of digital interfaces

GazePlotter has been particularly effective in studies analysing user interaction with layered or dynamic digital environments. In a study by Popelka, Komínek, and Vojtechovská (2024), it was used to visualise attention patterns on both static geological maps and interactive web maps. The dataset combined scanned images, screen recordings, and manually annotated gaze data from mobile eye-tracking glasses. GazePlotter’s ability to handle plain CSV input and render heterogeneous stimuli within a unified dashboard enabled flexible comparative visualisation. In a separate evaluation of COVID-19 dashboards (Porti Suarez & Popelka, 2023), a BeGaze-based workflow was replicated in GazePlotter, producing clearer and more interactive outputs. The illustrative walkthrough (Popelka et al., 2022) highlights a particularly impactful case in which participant-level sequence graphs—originally assembled through hours of manual editing—were reproduced in minutes using automated AOI assignment and dynamic visibility layers.

#### Education and didactics

GazePlotter has supported educational research by enabling sequence-level analysis of student attention. Skrabankova, Popelka, Beitlova, & Beitlova (2020) investigated how secondary school students interpreted physics graphs, using data recorded with GazePoint and processed through OGAMA. As GazePoint Analysis and OGAMA lacked support for scarf plots or advanced temporal visualisation, GazePlotter was used to generate interactive sequence graphs, replacing earlier workflows based on manually formatted Excel cells. In an unpublished chemistry education study, the tool was applied to analyse how students engaged with instructional videos demonstrating laboratory procedures. Visualising temporal gaze sequences revealed how attention shifted between equipment, procedural steps, and safety cues. In both cases, examining attention as a sequence—rather than as aggregate metrics—exposed patterns in comprehension and task engagement that would otherwise remain hidden.

#### Field-based studies

GazePlotter has also been applied in real-world environments to analyse gaze behaviour beyond laboratory settings. In a museum study (Popelka & Vyslouzil, 2025), researchers used Tobii Pro Glasses 3 to examine how visitors engaged with a geographic exhibit. While Tobii Pro Lab supported dynamic AOI assignment, it lacked scarf plot visualisation, which was instead carried out in GazePlotter without requiring custom scripts. Another field study—currently unpublished—investigated how tourists navigated urban space using printed USE-IT Europe maps while wearing mobile eye-trackers. The data were processed in GazePlotter to explore spatial referencing strategies and route-following behaviour. The commissioning institution received the outputs as preconfigured, shareable dashboards in JSON format.

#### Decision-making and imaginative cognition

Studies on abstract reasoning and symbolic interpretation have also employed GazePlotter to examine cognitive processes such as narrative engagement and visual decision-making. In religious science research, Bubík et al. (2023) visualised reading patterns in stories depicting in-group and out-group characters, mapping fixations to specific narrative segments to reveal implicit attentional biases. Another unpublished study on religious imagination explored how participants selected one image from a twelve-image grid to represent abstract concepts such as “goodness.” Dwell distributions and scanpaths across AOIs were visualised in GazePlotter, supporting the analysis of semantic preference formation through temporal gaze dynamics.

#### Non-eye-tracking sequential data

Additionally, GazePlotter’s support for custom CSV inputs enables the visualisation of arbitrary sequential data regardless of its origin. Beitlova (2024) used this feature to perform a structural content analysis of school world atlases. In this approach, individual atlas editions were treated as pseudo-participants, while the linear, page-by-page editorial sequence served as the timeline. Editorial elements—including map types (thematic, physical, or political), text blocks, charts, tables, and chapter themes—were encoded as AOIs to generate scarf plots that reveal the rhythm and density of information distribution across different publications. This use case confirms that GazePlotter can function as a general-purpose sequence analysis engine for any ordinal dataset.

## Discussion

GazePlotter treats scarf plots as instruments for first-pass interpretation rather than as static final figures. Rapid upload, participant/stimulus filtering, timeline switching (absolute, relative, ordinal), and side-by-side views can be performed directly in the browser, enabling researchers to explore temporal structure within minutes—without scripting. In Popelka et al. (2022), assembling participant-level sequences with dynamic AOIs (valuable for video stimuli, interactive interfaces, or field-based deployments) required hours of manual work; the replication took minutes.

The platform’s automatic parsing of six proprietary formats and CSV layouts—in contrast to existing scarf plot tools that typically support only one format or require manual preprocessing (Chen et al., 2023; Wu & Munzner, 2014; Yang & Wacharamanotham, 2018)—enables direct inspection of raw exports across heterogeneous software ecosystems. Local, client-side computation addresses privacy constraints in clinical, developmental, or institutional contexts, where gaze data—known to reveal sensitive personal attributes (Kröger et al., 2020)—must remain within trusted environments. Exported dashboards retain the full analytic state—including filters, layout, and software version—enabling peers to reopen and verify the exact configuration, and supporting reproducibility beyond what static figures allow.

Validation results indicate AOI-level metrics match BeGaze and Tobii within ±1 ms, and datasets over 12 GB are parsed entirely in-browser with modest memory use. These results were obtained on a mid-range laptop; lower-end devices also completed parsing, albeit more slowly. Together, these results indicate that web-based local computation can deliver both exploratory insight and quantitatively reliable outputs at scale. However, while GazePlotter visualizes group-level metrics via bar plots, detailed per-participant statistics are accessed via CSV export rather than in-tool tables, in contrast to other tools that provide integrated in-app statistics.

User evaluation ($N = 35$) was anchored by a remote, built-in instrumented protocol, which ensured that subsequent UEQ-S ratings (scale $-3.0$ to $+3.0$; Schrepp et al., 2017) followed verified functional task execution. Both dimensions yielded positive results: the “Excellent” Hedonic Quality ($M = 1.77$) places GazePlotter within the top 10% of global benchmarks, while “Above average” Pragmatic Quality ($M = 1.39$) indicates operational utility. Despite positive baseline scores, the lower clarity ($M = 1.0, SD = 1.7$) reveals localized friction, particularly on smaller screens—specifically, the absence of workspace zoom and indicators for off-screen elements—which suggests the need for navigational scaffolding to mitigate viewport-induced cognitive load. Perceived efficiency ($M = 1.9$) and user interest ($M = 2.0$) characterize the modular dashboard as an effective workspace for the rapid exploratory analysis of complex gaze sequences.

However, these benefits come with corresponding responsibilities. The flexibility afforded by real-time filtering and layout control increases researchers’ freedom and may introduce interpretive bias if not transparently documented. We therefore encourage sharing dashboard files or explicitly reporting analytic configurations when publishing results. Moreover, no-code tools may risk users overlooking analytic detail. The tool is therefore best positioned as a scaffold for initial understanding, supporting early exploration before committing to formal inspection or modelling. Crucially, the validity of any AOI-based visualisation—whether exploratory or final—depends on clearly defined and methodologically sound AOIs (Hessels et al., 2016; Orquin et al., 2016). No visualisation tool can compensate for poorly constructed segmentation schemes or inconsistent labelling practices.

While the AOI-centric focus defines the tool’s current analytical boundaries—reflecting the pragmatic development constraints of a single-developer project—this same discretisation framework enables methodological transfer. By abstracting units as pseudo-AOIs and entities as pseudo-participants (Beitlova, 2024), GazePlotter can quantify the rhythm and density of categorical ordinal sequences derived from non-eye-tracking data, such as media content.

Looking ahead, future work will bridge the observed clarity variance ($SD = 1.7$) by providing an integrated interactive walkthrough for first-time users and improving workspace navigation, thereby mitigating viewport-induced cognitive load. Further, future extensions include broadening visualisation types, input compatibility (e.g., EyeLink), and exploring a machine-learning-based pattern discovery. The modular, open-source codebase enables these capabilities to grow beyond our own roadmap, allowing researchers to fork, adapt, or contribute new components as analytical needs diversify. GazePlotter is not a finished product but a scaffold for building and iterating on open, inspectable workflows—aligned with broader calls for cumulative, transparent scientific infrastructure (Burgelman et al., 2019).

## Conclusion

GazePlotter offers a lightweight, browser-based platform for exploring temporally structured gaze data through scarf plots and related visualisations. By automating AOI-based sequence extraction across multiple proprietary formats and supporting local, privacy-preserving analysis, the tool addresses long-standing usability and interoperability barriers in eye-tracking research. Exportable dashboards preserve the full analytic state—including filters, layout, and settings of visualisation panels, and parsing metadata—promoting reproducibility, transparent reporting, and reliable exploratory use. User evaluations indicate positive user experience ratings, while highlighting clarity-related constraints that guide ongoing refinement. Rather than serving as a final analysis endpoint, GazePlotter is intended for exploratory inspection, hypothesis refinement, and collaborative workflows—bridging the gap between raw gaze data and interpretable visual narratives, while remaining dependent on methodologically sound AOI definitions.

## Supplementary Information

Below is the link to the electronic supplementary material.

Supplementary file 1 (zip 19237 KB)

## Data Availability

Data from case studies are available in the GazePlotter Sample Data repository,https://doi.org/10.17605/OSF.IO/XD3ZV (Vojtechovska & Popelka, 2025). Any request for additional eye-tracking data should be made to the corresponding author.

## Footnotes

1 https://github.com/misavojte/GazePlotter

## Code Availability

The source code for both GazePlotter and its guide is available on GitHub under the GNU General Public Licence v3.0: $\bullet$ https://github.com/misavojte/GazePlotter$\bullet$ https://github.com/misavojte/GazePlotterDocs

## Declarations

### Generative large language models use

Generative large language models were used for multiple purposes during the creation of GazePlotter and this article. For some paragraphs of this paper, we employed ChatGPT 4o to rephrase statements, merge them, or scaffold them from multi-lingual bullet points. In addition, the developer used GitHub Copilot for code suggestions in later development phases. These tools were used for efficiency in drafting and interface development, with all conceptual and methodological decisions made by the authors.
