# Supplementary material for: GazePlotter: An open-source solution for the automatic generation of scarf plots from eye-tracking data
Source: Behav Res Methods. 2026 Mar 12;58(3):85. doi: 10.3758/s13428-026-02959-5 (PMC12982204; doi:10.3758/s13428-026-02959-5)
Supplement: Supplementary file 1 — (zip 19237 KB) [file 13428_2026_2959_MOESM1_ESM.zip › 13428_2026_2959_MOESM1_ESM/C_GazePlotter_1-7-1_Code_Build.zip!/build/etvis.html]

GazePlotter | Free eye-tracking data visualisation via scarf plots


GazePlotter Guide

 

# GazePlotter

## Free eye-tracking data visualisation

Transform eye gaze data from eye trackers to interactive scarf plots.
No registration, no ads and no data stored on a server. We
love open science.

Instructions for the ETRA participants (UX evaluation)

On your computer, before starting the form:

1. Switch the stimulus in the Scarf Plot.

2. Duplicate the scarf plot, move it in the dashboard, and assign different participant subgroups to each.

3. Change the Aggregation method of the Bar Plot and duplicate it.

4. Customise the current Areas of Interests (AOIs) in the data. Assign two AOIs into one group, change its displayed color and see the effects in the dashboard.

Fill out questionnaire

Import workspace or data  Reload ETVIS data Export workspace or data

## Processing data

Please wait while we prepare your visualizations. This may take a moment
depending on the size of your dataset.

## Open source Svelte library

GazePlotter is an open source library, written in Svelte and
TypeScript. You can use it for free or modify its code to fit your
specific needs in your projects.

See GitHub repo

## Works with Tobii, SMI & more

The app creates interactive sequence charts and other analysis from
Tobii, SMI, GazePoint and other eye trackers. Just upload your data
and see the results.

Which files to upload?

## Interactive scarf plots

Scarf plots (sequence charts) are a great way to visualise
eye-tracking data. They show the order of fixations in time and their
hits on areas of interest (AOI). In GazePlotter, they are interactive,
customisable and easy to share.

## Runs without Internet

GazePlotter does not store your data on a server, thus ensuring data
privacy. All is done in your browser. You can use it on PCs, Macs,
tablets, even off-line!

How to download GazePlotter?

## Other eye tracking tools

Eye-Tracking Group at Department of Geoinformatics, Palacký University
Olomouc, develops other eye tracking tools for free use.

More eye tracking tools

 

GazePlotter, version 1.7.1 (August 5, 2025)

Coded & designed by Michaela Vojtěchovská, idea by Stanislav Popelka

Department of Geoinformatics, Palacký University Olomouc
