# Supplementary material for: GazePlotter: An open-source solution for the automatic generation of scarf plots from eye-tracking data
Source: Behav Res Methods. 2026 Mar 12;58(3):85. doi: 10.3758/s13428-026-02959-5 (PMC12982204; doi:10.3758/s13428-026-02959-5)
Supplement: Supplementary file 1 — (zip 19237 KB) [file 13428_2026_2959_MOESM1_ESM.zip › 13428_2026_2959_MOESM1_ESM/C_GazePlotter_1-7-1_Code_Source.zip!/GazePlotter-main/src/app.html]

%sveltekit.head%


%sveltekit.body%
