# Supplementary material for: GazePlotter: An open-source solution for the automatic generation of scarf plots from eye-tracking data
Source: Behav Res Methods. 2026 Mar 12;58(3):85. doi: 10.3758/s13428-026-02959-5 (PMC12982204; doi:10.3758/s13428-026-02959-5)
Supplement: Supplementary file 1 — (zip 19237 KB) [file 13428_2026_2959_MOESM1_ESM.zip › 13428_2026_2959_MOESM1_ESM/D_GazePlotter_Survey_UEQS_Analysis.zip!/GazePlotterUEQ-main/ueq_results_figure.pdf]

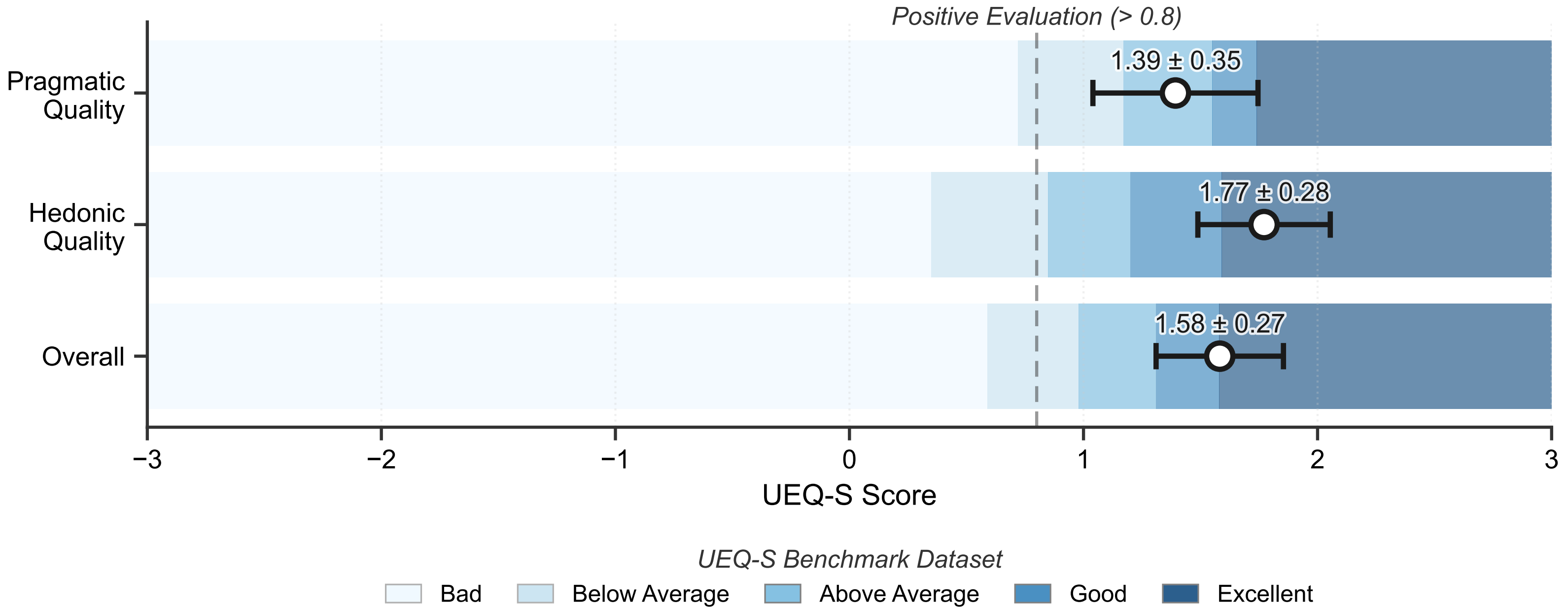
Positive Evaluation (> 0.8)
Pragmatic Quality
Hedonic Quality
Overall
1.39 ± 0.35
1.77 ± 0.28
1.58 ± 0.27
−3
−2
−1
0
1
2
3
UEQ-S Score
UEQ-S Benchmark Dataset
Bad
Below Average
Above Average
Good
Excellent
